# Dietary pyrroloquinoline quinone and spermidine in healthy longevity: targeting the hallmarks of aging

**DOI:** 10.3389/fragi.2026.1791853

**Published:** 2026-05-15

**Authors:** Tomoe Numaguchi, Mai Nakamura, Tomoyo Koshizawa, Nur Syafiqah Mohamad Ishak, Katsuyuki Hashimoto

**Affiliations:** Niigata Research Laboratory, Mitsubishi Gas Chemical Company, Inc., Niigata, Japan

**Keywords:** anti-aging, autophagy, longevity, mitochondria, nutritional intervention, pyrroloquinoline quinone, spermidine

## Abstract

**Background:**

Aging is a multifaceted biological process driven by interconnected cellular and molecular hallmarks. As geroscience increasingly prioritizes healthspan over lifespan, nutritional interventions targeting multiple aging mechanisms have gained attention as accessible strategies to mitigate age-related functional decline.

**Objective:**

This mini review synthesizes recent evidence on how the bioactivities of two food-derived geroprotective compounds, pyrroloquinoline quinone (PQQ) and spermidine (SPD), intersect with the hallmarks of aging and their distinct and overlapping roles in maintaining cellular homeostasis.

**Findings:**

PQQ primarily functions as a mitochondrial and redox regulator, enhancing mitochondrial biogenesis and bioenergetic capacity through the AMP-activated protein kinase (AMPK) and sirtuin1 (SIRT1)/peroxisome proliferator-activated receptor gamma coactivator 1-alpha pathways. In contrast, SPD acts as a key regulator of cellular quality control by inducing macroautophagy and preserving proteostasis, largely through modulation of histone and autophagy-related protein acetylation. These complementary mechanisms converge on several key hallmarks of aging, including genomic instability, deregulated nutrient sensing, mitochondrial dysfunction, and chronic inflammation.

**Conclusion:**

The anti-aging mechanisms of PQQ and SPD originate from distinct upstream biochemical processes but converge on shared signaling hubs, including the AMPK/SIRT1 axis and autophagy-related networks. This convergence suggests a coordinated network-level complementarity that may offer a more robust intervention against age-related decline than targeting independent pathways alone.

## 1 Introduction

Aging is no longer viewed as an inevitable singular physical decline but rather as a coordinated cascade of interconnected biological “hallmarks,” including genomic instability, telomere attrition, epigenetic alterations, and chronic inflammation (López-Otín et al., 2023). With the rapidly shifting global demography, the primary objective of geroscience has shifted from merely extending lifespan (total years lived) to expanding healthspan—the period of life spent in good functional health (Melov, 2016; Wang et al., 2025). Nutritional interventions targeting multiple interconnected aging hallmarks have emerged as accessible and potentially safe strategies to mitigate age-related functional decline. However, the expanding longevity supplement market requires the establishment of rigorous mechanistic frameworks to distinguish evidence-based interventions from speculative or weakly validated approaches (Kaufman et al., 2025).

Among candidate nutritional geroprotectors, the bioactive compounds pyrroloquinoline quinone (PQQ) and spermidine (SPD) have gained increasing attention owing to their pleiotropic effects on cellular aging pathways. SPD is a ubiquitous dietary polyamine and potent inducer of autophagy and proteostasis. Accumulating evidence, including human studies, suggests that SPD intake benefits cardiovascular and cognitive function (Eisenberg et al., 2009; Madeo et al., 2010; 2018; Schroeder et al., 2021). Conversely, PQQ is a redox-active micronutrient thought to function as a vitamin-like compound based on its essential role in mitochondrial development, growth, and reproductive performance in animal models. In preclinical studies, PQQ was found to regulate mitochondrial biogenesis, oxidative balance, and energy-sensing pathways (He et al., 2003; Itoh et al., 2016; Jonscher et al., 2021). Importantly, early human intervention studies using PQQ disodium salt reported beneficial effects on brain function, cognitive performance, and physiological stress responses (Ikemoto et al., 2024), supporting its translational relevance for managing healthy aging.

Although often examined separately, these compounds represent a dual-action framework involving distinct upstream biological processes that converge on shared cellular maintenance pathways: PQQ primarily supports cellular energy generation through mitochondrial biogenesis, whereas SPD promotes the maintenance and quality control of existing cellular machinery. Accordingly, this mini review synthesizes recent evidence (2020–2025) in terms of aging hallmarks through a structured literature search using PubMed, Scopus, and Web of Science, applying search terms including “pyrroloquinoline quinone,” “spermidine,” “aging,” “mitochondria,” “autophagy,” and related keywords. Detailed search criteria are provided in the Supplementary Material.

We focused on the evolution of geroscience from descriptive observations to mechanistic interpretation, highlighting how PQQ and SPD have progressed from general redox or autophagic modulators to defined signaling regulators. By delineating their shared and distinct molecular targets, we aim to clarify their relevance within integrated nutritional strategies for promoting healthy longevity.

## 2 Dietary sources and food ingredients

### 2.1 PQQ

PQQ is found in various plant-based foods, including spinach, green peppers, and parsley, as well as in animal-derived products, such as milk and eggs. Notably, fermented foods, including tofu, miso, and natto, contain high concentrations of PQQ (up to 61 ng/g in natto) (Kumazawa et al., 1995). Despite its prevalence, typical dietary PQQ concentrations remain relatively low (around 1–30 ng/g across most foods) (Noji et al., 2007).

**FIGURE 1:**
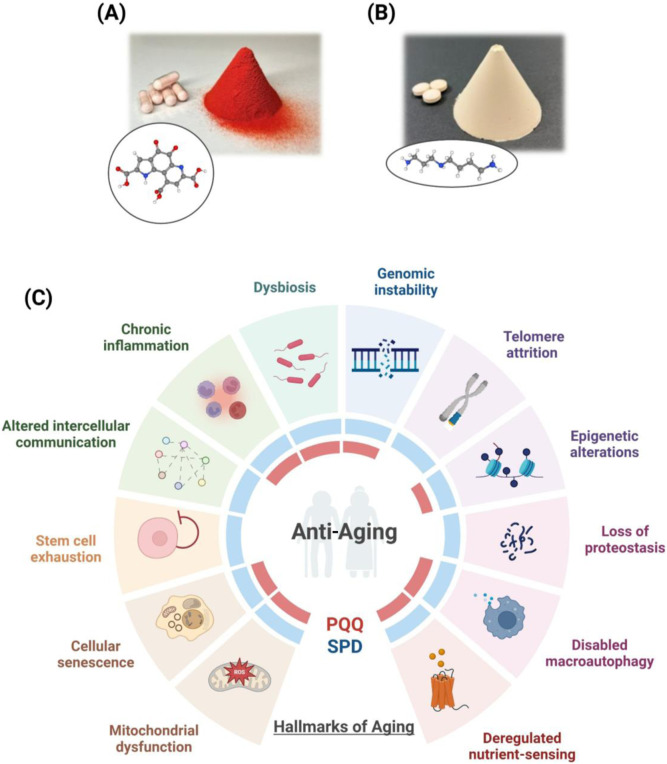
Modulatory effects of pyrroloquinoline quinone (PQQ) and spermidine (SPD) on biological hallmarks of aging. **(A)** Chemical structure and representative physical form of PQQ disodium salt. PQQ acts as a redox-active micronutrient that supports mitochondrial biogenesis, redox balance, and bioenergetic regulation. **(B)** Chemical structure and representative form of high-SPD-producing yeast preparation used for dietary intake. Yeast-derived SPD provides a natural polyamine matrix that supports cellular homeostasis, primarily through the activation of macroautophagy and related quality-control pathways. **(C)** Schematic overview of the 12 hallmarks of aging and biological domains modulated by PQQ and SPD. The outer ring depicts the primary, antagonistic, and integrative hallmarks that contribute to age-associated functional decline. The inner color-coded rings indicate the hallmarks most prominently influenced by PQQ (red) and SPD (blue), based on evidence from cellular, animal, and emerging human studies. Figure created using BioRender.com.

To enable higher and more consistent intake, commercial BioPQQ is produced as a functional food ingredient via microbial fermentation using a non-genetically modified strain of *Hyphomicrobium denitrificans* (CK-275), with an established regulatory profile (Figure 1A). Regulatory and safety evaluations have supported its use as a dietary ingredient. PQQ is recognized as a New Dietary Ingredient in the United States and authorized as a Novel Food in the European Union following safety assessment by EFSA, facilitating broader use in supplements and functional foods (Turck et al., 2017). Multiple reviews have also summarized the safety profile and “vitamin-like” biological relevance of PQQ in mammalian systems, particularly in mitochondrial and redox biology (Jonscher et al., 2021; Charrier et al., 2024; Ikemoto et al., 2024; Yan et al., 2024).

### 2.2 SPD

SPD is a naturally occurring polyamine with significantly higher dietary concentrations than PQQ, typically ranging from tens to hundreds µg/g and is abundant in whole grains, legumes, soybeans, mushrooms, and aged cheeses (Atiya Ali et al., 2011). Among dietary sources, natto is a rich source of both PQQ and SPD, emphasizing the potential relevance of fermented soy products in nutritional strategies for healthy aging. Epidemiological studies have reported strong associations between higher dietary SPD intake and reduced all-cause mortality, as well as improved cardiovascular health (Soda et al., 2009).

Beyond whole foods, SPD is also produced as a food ingredient using specialized microbial fermentation processes. High-SPD yeast products derived from *Saccharomyces cerevisiae* have been developed by optimizing fermentation conditions to enhance endogenous polyamine biosynthesis without genetic modification (Koshizawa et al., 2025). These standardized yeast-derived ingredients provide a scalable and stable source of SPD for dietary supplementation, with potential utility in promoting autophagy and supporting cellular homeostasis (Figure 1B).

## 3 How PQQ and SPD target the hallmarks of aging

PQQ and SPD exert pleiotropic biological effects that intersect with multiple hallmarks of aging (Figure 1C; Table 1). Evidence from *in vitro*, *in vivo*, and human studies suggests that PQQ primarily acts as a mitochondrial and redox modulator (Jonscher et al., 2021; Ikemoto et al., 2024; Yan et al., 2024), whereas SPD predominantly induces autophagy and cellular turnover (Alsaleh et al., 2020; Hofer et al., 2024).

**TABLE 1:** Summary of evidence (2020–2025) linking PQQ and SPD to key hallmarks of aging and underlying mechanisms.

Hallmark of aging	Key effects & mechanisms	References
Genomic instability	PQQ: • Reduces ROS and oxidative DNA lesions • Supports genome integrity under oxidative stress SPD: • Reduces DNA damage via antioxidant effects • Enhances autophagy-linked genome protection • Supports DNA repair capacity	PQQ: Li et al. (2022) SPD: Coeli-Lacchini et al. (2023)
Telomere attrition	PQQ: • Direct evidence limited SPD: • Reduces oxidative telomere damage • Enhanced autophagy-mediated protection • Preserves telomere length	SPD: Wirth et al. (2021)
Epigenetic alterations	PQQ: • Inhibits PRC2/EZH2 activity • Modulates sirtuin-associated chromatin regulation SPD: • Regulated gene expression via polyamine metabolism • Modulates acetylation-dependent pathways • Influence RIPK-1-related signaling	PQQ: Jiang M. et al. (2025) SPD: Kojić et al. (2024), Zhang T. et al. (2024)
Loss of proteostasis	SPD: • Activates autophagy-mediated protein quality control • Support organelle turnover under stress • Induces autophagy via mTORC1-independent routes	SPD: Xie et al. (2025), Hofer et al. (2024)
Disabled macroautophagy	PQQ: • Increases autophagy in *C. elegans* and mammalian models • Activates PINK1/Parkin-associated mitophagy • Restores cardiolipin-dependent mitophagy under metabolic stress SPD: • Potent inducer of autophagy • Regulates acetylation-dependent autophagy pathways • Restores autophagy in T-cells	PQQ: Gao et al. (2021), Yang et al. (2021), Zhang et al. (2020), Sagar et al. (2023) SPD: Alsaleh et al. (2020), Schwarz et al. (2020); Schroeder et al. (2021), Pekar et al. (2021), Zhang et al. (2023), Díaz-Osorio et al. (2025)
Deregulated nutrient sensing	PQQ: • Activates AMPK signaling • Enhances SIRT1–PGC-1α metabolic pathways SPD: • Improves metabolic dysfunction (e.g., steatosis/inflammation) • Required for fasting-mediated autophagy/longevity	PQQ: Cheng et al., 2020; Supruniuk et al., 2020; Yang et al., 2021 SPD: Liao et al., 2021; Hofer et al., 2024
Mitochondrial dysfunction	PQQ: • Enhances mitochondrial function and ATP production • Improves redox balance • Promotes mitochondrial biogenesis SPD: • Preserves mitochondrial function via eIF5A axis • Improves membrane potential and ATP production • Associated with reduced brain aging indicator • Upregulates SIRT1-PGC-1α-dependent pathways	PQQ: Gao et al. (2021), Gao et al. (2022), Liu et al., 2024 SPD: Wang et al., 2020; Messerer et al. (2023), Ueno et al. (2023), Szabo et al. (2024)
Cellular senescence	PQQ: • Suppresses SASP and inflammatory signaling • Exhibits senomorphic effects • Delays aging phenotypes SPD: • Mitigates senescence-associated dysfunction • Associated with reduced brain aging indicators • Improves immune and tissue regeneration	PQQ: Li et al. (2022), Li et al. (2023), Liu et al. (2024), Mohamad Ishak et al. (2024), Jiang B. et al. (2025), Liu et al. (2025) SPD: Niu et al. (2023), Singh et al. (2025), Yoshida et al. (2025)
Stem cell exhaustion	PQQ: • Direct evidence limited SPD: • Promotes stem cell activation (e.g., muscle satellite cells) • Regulates translation via eIF5A axis • Supports mitophagy in progenitor cells	SPD: Kasahara et al. (2024), Zhang Q. et al. (2024), Zhou et al. (2025)
Altered intercellular communication	PQQ: • Modulates immune cell population (single-cell evidence) SPD: • Enhances immune cell function via autophagy • Regulates macrophage polarization • Rebalances inflammatory signaling networks	PQQ: Liu et al. (2024) SPD: Huang et al. (2023), Díaz-Osorio et al. (2025)
Chronic inflammation (inflammaging)	PQQ: • Suppresses NF-κB signaling • Reduces pro-inflammatory cytokines SPD: • Exerts systemic anti-inflammatory effects • Modulates immune responses via autophagy • Reduces neuro- and metabolic inflammation	PQQ: Wang et al. (2019), Mohamad Ishak et al. (2024) SPD: Xu et al. (2020), Ma et al. (2021), Freitag et al. (2022), Ou et al. (2024)
Dysbiosis (gut–immune axis)	PQQ: • Modulates gut microbiota composition • Increases SCFA production • Supports intestinal barrier function SPD: • Improves gut barrier integrity • Restores microbiota balance • Reduces inflammation and metabolic dysfunction	PQQ: Huang et al. (2020) SPD: Ma et al. (2020), Liu et al. (2022), Niechcial et al. (2023)

The available literature supports the interconnected modulation of aging pathways by PQQ and SPD rather than them acting on isolated molecular targets. Although PQQ and SPD are often described as mechanistically distinct, many of their downstream effects converge on common signaling hubs, including AMPK, SIRT1, and autophagy-related pathways (Cheng et al., 2020; Hofer et al., 2024). This overlap does not indicate redundancy but reflects the integration of distinct upstream metabolic signals into shared regulatory networks that govern cellular homeostasis (Supplementary Table S1). In the following sections, we examine their roles across individual hallmarks of aging, integrating mechanistic and clinical evidence within a narrative framework.

### 3.1 Genomic instability: redox protection and DNA repair support by PQQ and SPD

Genomic instability is a hallmark of aging, driven by the accumulation of DNA damage, reduced repair capacity, and chronic oxidative stress (López-Otín et al., 2023). Both PQQ and SPD help preserve genomic integrity through complementary mechanisms that limit oxidative DNA damage and support DNA repair (Li et al., 2022; Coeli-Lacchini et al., 2023). In B-lymphoma Mo-MLV insertion region 1 homolog (Bmi-1)-knockout mice, PQQ supplementation significantly attenuated oxidative stress-induced DNA damage, as evidenced by reduced levels of 8-hydroxy-2′-deoxyguanosine (8-OHdG), a widely used biomarker of oxidative DNA lesions (Li et al., 2022). This protective effect is largely attributed to the ability of PQQ to suppress reactive oxygen species (ROS) activity, thereby limiting oxidative damage and supporting DNA integrity and repair capacity (Jonscher et al., 2021).

SPD has also been reported to reduce genotoxic stress and support DNA damage responses in disease models. In an oral carcinogenesis model, SPD suppressed DNA damage while promoting protective cellular responses, including autophagy induction and improved DNA damage handling (Coeli-Lacchini et al., 2023). Broadly, dietary polyamines have been considered as modifiers of age-related genome maintenance, though the strength and directness of evidence vary by model and endpoint (Soda, 2022).

### 3.2 Telomere attrition: indirect preservation via oxidative stress reduction and autophagy

Telomere attrition reflects the progressive shortening and functional impairment of chromosomal ends, contributing to replicative senescence and cellular aging (López-Otín et al., 2023). Despite limited direct evidence for telomere regulation by PQQ, SPD appears to support telomere integrity through indirect mechanisms involving oxidative stress reduction and autophagic homeostasis enhancement. Wirth et al. (2021) demonstrated that SPD supplementation attenuated telomere damage by lowering cellular oxidative stress and activating autophagy, supporting overall cellular homeostasis. These effects were associated with telomere length preservation and may contribute to preventing premature telomere shortening and age-related functional decline. Another study has linked the antioxidant properties and calorie restriction-like effects of SPD to improved telomere maintenance (Hofer et al., 2024). Collectively, evidence suggests that SPD may attenuate telomere shortening and support telomere stability by alleviating cellular stress and promoting autophagy; however, further mechanistic studies and long-term human trials are needed.

### 3.3 Epigenetic alterations: polyamine- and sirtuin-mediated chromatin regulation

Age-associated epigenetic alterations, including changes in histone modifications, DNA methylation, and chromatin architecture, disrupt essential gene expression programs for maintaining cellular homeostasis (López-Otín et al., 2023). Emerging evidence indicates that both PQQ and SPD modulate epigenetic regulation through distinct yet convergent mechanisms involving sirtuin signaling and polyamine-dependent chromatin remodeling. In human B-cell lymphoma models, PQQ interacted with epigenetic regulators by inhibiting the activity of polycomb repressive complex 2 (PRC2) [enhancer of zeste homolog 2 (EZH2)] methyltransferase and modulating the expression and activity of sirtuins SIRT1 and SIRT3 (Jonscher et al., 2021, Jiang M. et al., 2025). These findings suggest that PQQ may influence chromatin structure and downstream gene expression programs relevant to aging and pathological contexts such as cancer and developmental dysregulation.

SPD exerts epigenetic effects primarily through its role in polyamine metabolism and chromatin modification. SPD decreases histone H3 acetylation, thereby altering chromatin accessibility and inducing autophagy, which contributes to improved cellular function in senescent cells and suppression of inflammatory signaling (Satarker et al., 2024; Kojić et al., 2024). Furthermore, fluctuations in polyamine metabolism and the S-adenosyl methionine pool can influence epigenetic states, including DNA methylation patterns, involved in metabolic homeostasis and longevity (Tain et al., 2020; Soda, 2020). Recently, SPD has been suggested to modulate inflammatory and regulated cell death pathways through acetylation-dependent regulation of receptor-interacting protein kinase 1 (RIPK1), with potential implications for the suppression of metabolic disease progression (Zhang T. et al., 2024). Although consistent in animal models, further studies are needed to assess tissue specificity and long-term relevance in humans.

### 3.4 Loss of proteostasis: autophagy-dependent maintenance of protein and organelle quality

Loss of proteostasis involves impaired protein synthesis, folding, and degradation, leading damaged proteins and organelles accumulating during aging (López-Otín et al., 2023). SPD has emerged as a potent modulator of proteostasis, which is progressively compromised by aging and cellular stress across multiple organisms. Recent reviews highlighted the role of SPD in promoting healthy aging through the maintenance of mitochondrial function and metabolic regulation mediated by small-molecule pathways (Qin et al., 2024). Mechanistically, SPD facilitates clearance of damaged proteins and dysfunctional mitochondria by inducing autophagy (Hofer et al., 2024; Xu et al., 2020). Recent evidence indicated that endogenous SPD levels increase during intermittent fasting or calorie restriction, which is essential for the induction of autophagy, thereby contributing to lifespan extension (Hofer et al., 2024). These findings support a functional role for SPD in mitigating age-associated loss of proteostasis.

### 3.5 Disabled macroautophagy: enhancement of autophagic and mitophagic pathways by PQQ and SPD

Impaired macroautophagy drives aging by promoting proteotoxic stress, mitochondrial dysfunction, and chronic inflammation (López-Otín et al., 2023). Both PQQ and SPD improved autophagy-related cellular quality control in multiple experimental models.

In *Caenorhabditis elegans*, PQQ supplementation extended lifespan through activation of macroautophagy, as evidenced by an increased expression of autophagy-related genes such as *lgg-1* and *bec-1*, along with enhanced formation of GFP::LGG-1 puncta. Importantly, this autophagic activation was dependent on insulin and insulin-like growth factor 1 (IGF-1) signaling and was required for the longevity-promoting effects of PQQ (Yang et al., 2021). In mammalian microglial injury models, PQQ enhanced autophagy-associated markers in LPS-stimulated HAPI microglial cells (Gao et al., 2021) and suppressed rotenone-induced microglial inflammation through autophagy enhancement (Zhang et al., 2020). Mechanistically, PQQ increased autophagy/mitophagy-associated markers (e.g., LC3-II/LC3-I ratio, Atg5) and supported mitochondria–lysosome co-localization consistent with PINK1/Parkin-mediated mitophagy activation (Zhang et al., 2020). PQQ also restored impaired cardiolipin-dependent mitophagy in mesenchymal stem cells under obesity-associated metabolic stress (Sagar et al., 2023). In addition, PQQ promoted mitochondrial biogenesis and improved mitochondrial-related outcomes under neurotoxic stress conditions, supporting the close coupling between mitochondrial quality control and autophagy-dependent maintenance (Cheng et al., 2020).

SPD likewise improves autophagy-linked cellular maintenance. In experimental models, SPD promoted autophagy/mitophagy-related processes and delayed aging-associated phenotypes (Zhang et al., 2023), including in *C. elegans* via a mitophagy pathway involving PINK1/PDR-1 (Yang et al., 2020) and in mammalian aging contexts where SPD-induced hypusination preserved mitochondrial and cognitive function (Hofer et al., 2021). Recent work further demonstrated that SPD is required for fasting-mediated autophagy and the associated longevity benefits, supporting a physiological link between nutrient state and autophagy-dependent quality control (Hofer et al., 2024). In humans, observational and intervention studies reported associations between dietary SPD intake/supplementation and cognitive or brain-structure outcomes in older adults, providing translational context for dietary interventions in managing healthy aging (Schwarz et al., 2020; Pekar et al., 2021; Schroeder et al., 2021). Collectively, these findings support continued controlled clinical investigations, ideally including standardized autophagy-related biomarkers alongside functional endpoints.

### 3.6 Deregulated nutrient sensing: improvements to disrupted metabolic homeostasis

Dysregulation of nutrient-sensing pathways, including AMPK, sirtuins, and insulin/IGF-1 signaling, is closely linked to metabolic aging (López-Otín et al., 2023). In muscle cell models, PQQ increased AMPK phosphorylation and upregulated PGC-1α and SIRT1 expression, indicating enhanced cellular energy sensing and mitochondrial regulation under metabolic stress conditions (Cheng et al., 2020; Supruniuk et al., 2020; Charrier et al., 2024). In naturally aged mice, dietary PQQ supplementation prevented abnormal age-related fat loss and improved metabolic efficiency, suggesting preservation of adipose tissue function during aging (Mohamad Ishak et al., 2024). Although the precise molecular mechanisms are unclear, these effects are likely mediated through integrated AMPK–sirtuin–mitochondrial signaling networks. Additionally, lifespan extension observed in *C. elegans* supports a role for PQQ in modulating insulin/IGF-1 signaling within conserved nutrient-sensing pathways (Yang et al., 2021).

SPD ameliorates metabolic dysfunction across multiple tissues, including adipose tissue, liver, and intestines. In mouse models of overnutrition or high-fat diet feeding, SPD promotes lipolysis in visceral adipose tissue, thereby limiting fat accumulation. Regulation of fibroblast growth factor 21 (FGF21) expression in hepatic and adipose tissues mitigates insulin resistance, dyslipidemia, and hepatic steatosis (Liao et al., 2021). Under fasting or calorie-restricted conditions, endogenous SPD synthesis increases rapidly, enhancing autophagy through hypusination of eukaryotic translation initiation factor 5A (eIF5A) and contributing to lifespan and healthspan extension (Hofer et al., 2024). These findings suggest that SPD restores intracellular metabolic homeostasis disrupted by abnormal nutrient signaling, partially through coordinated autophagic activation and metabolic remodeling.

### 3.7 Mitochondrial dysfunction: biogenesis enhancement by PQQ and quality control by SPD

Mitochondrial dysfunction, a central hallmark of aging, is characterized by reduced bioenergetic capacity, increased oxidative stress, and impaired mitochondrial quality (López-Otín et al., 2023). PQQ primarily enhances mitochondrial biogenesis and redox balance, whereas SPD supports mitochondrial integrity through mitophagy and broad metabolic remodeling. In premature ovarian insufficiency mouse models, combined treatment with mesenchymal stem cell-derived mitochondria and PQQ significantly enhanced mitochondrial biogenesis via upregulation of SIRT1 and PGC-1α (Liu et al., 2024). Comparable protective effects were observed in auditory House Ear Institute–Organ of Corti 1 (HEI-OC1) cells, where PQQ restored ATP production, promoted mitochondrial biogenesis, and attenuated oxidative stress-induced premature aging (Gao et al., 2022). In microglial cell models, PQQ further reduced apoptosis by enhancing autophagic activity and modulating lysosomal distribution, underscoring the close interplay between mitochondrial quality control and autophagic processes in PQQ-mediated cytoprotection (Gao et al., 2021).

SPD also protects against mitochondrial dysfunction through multiple complementary mechanisms. In the myocardium, SPD activates mitochondrial biogenesis via the SIRT1–PGC-1α pathway, improving cardiac function in aging models (Wang et al., 2020). In experimental aging contexts, SPD supplementation has been linked to preserved mitochondrial and cognitive/brain-aging phenotypes (Liang et al., 2021; 2025). In human induced pluripotent stem cell (iPSC)-derived neurons, SPD enhanced mitochondrial respiratory capacity, membrane potential, and ATP production while reducing ROS levels, thereby improving neuronal energy homeostasis (Szabo et al., 2024). In addition, SPD has been reported to influence mitochondrial number and morphology in the aged heart (Messerer et al., 2023) and improve angiogenic capacity in senescent endothelial cells and neovascularization in aged mice (Ueno et al., 2023). Collectively, these findings suggest that SPD may mitigate age-related functional decline across multiple tissues, including the cardiovascular and central nervous systems.

### 3.8 Cellular senescence: suppression of senescence programs and senescence-associated secretory phenotype (SASP) signaling

Cellular senescence contributes to age-related tissue dysfunction through irreversible cell-cycle arrest and secretion of pro-inflammatory factors (López-Otín et al., 2023). PQQ may suppress senescence-associated pathways across multiple tissues. In Bmi1-deficient mice, PQQ inhibited skin aging by reducing oxidative DNA damage and suppressing matrix metalloproteinase activity (Li et al., 2022). *In vitro*, PQQ attenuated DNA damage-induced senescence by upregulating SIRT1 and inhibiting ATM/p53 signaling in granulosa and stromal cell models (Liu et al., 2024). Notably, PQQ also suppressed pro-inflammatory features of senescent cells and reduced SASP-related phenotypes in human cell models (Jiang B. et al., 2025). PQQ preserved immune regenerative capacity, prevented age-related bone loss via Nrf2-linked stress responses, and suppressed senescence-associated phenotypes in other experimental systems, collectively highlighting its broad anti-senescent potential (Li et al., 2023).

SPD attenuates cellular aging through complementary mechanisms, including reduction of oxidative stress, activation of autophagy, and improvement of mitochondrial and metabolic homeostasis across multiple experimental models (Yuan et al., 2021; Niu et al., 2023; Zimmermann and Madeo, 2023). Oxidative stress and disruption of ionic homeostasis were corrected in both d-galactose-induced and naturally aged rat models following SPD supplementation, improving age-associated functional outcomes (Singh et al., 2025). In human studies, higher circulating SPD levels were associated with lower levels of brain-aging indicators (Wortha et al., 2023), supporting a potential role for SPD as a geroprotective factor linked to polyamine metabolism.

### 3.9 Stem cell exhaustion: polyamine-mediated protection

Stem cell exhaustion, marked by reduced self-renewal and regenerative capacity, impairs tissue maintenance and repair during aging (López-Otín et al., 2023). Preclinical studies suggest that SPD may suppress stem cell depletion across multiple experimental models. In muscle stem cells, SPD promoted stem cell activation and regenerative responses via eIF5A-dependent translational control (Zhang Q. et al., 2024). In a diabetic periodontitis model, SPD attenuated senescence-associated phenotypes in periodontal ligament stem cells, with mechanistic links to enhanced mitophagy (Zhou et al., 2025). Additionally, dietary polyamine intake promoted the proliferation of intestinal epithelial stem cells and facilitated intestinal adaptation in a short bowel syndrome model (Kasahara et al., 2024). These findings suggest a potential role of SPD and/or dietary polyamines in maintaining stem cell-related homeostasis in aging-relevant contexts; however, direct evidence in humans remains limited.

### 3.10 Altered intercellular communication: immune and inflammatory network

Altered intercellular communication during aging involves disrupted immune signaling, inflammation, and impaired tissue coordination (López-Otín et al., 2023). Single-cell transcriptomic analysis indicated that PQQ remodels the immune-aging landscape in the hematopoietic/immune system, consistent with broader effects on age-associated immune dysregulation and intercellular signaling (Liu et al., 2025). SPD may partially restore communication by promoting autophagy-linked immune homeostasis and mitochondrial quality control. SPD improved immune-related phenotypes by inducing anti-inflammatory (M2-like) macrophage polarization and restoring autophagic function in T cells (Liu et al., 2020; Alsaleh et al., 2020). In cellular transport disorders, SPD normalized cellular function by compensating for underlying autophagy deficiencies (Díaz-Osorio et al., 2025). Furthermore, SPD enhanced mitophagy- and antioxidant-associated responses in vascular endothelial and mesenchymal stromal cells, consistent with improved stress resilience and reduced pro-inflammatory features (Huang et al., 2023; Ueno et al., 2023). Collectively, these findings support the role of SPD in mitigating age-associated alterations in intercellular communication, particularly inflammation and immune imbalance, through coordinated regulation of autophagy and mitochondrial quality control mechanisms.

### 3.11 Chronic inflammation: nuclear factor kappa B (NF-κB) suppression and immune homeostasis

Chronic low-grade inflammation, often referred to as “inflammaging,” substantially contributes to the development and progression of age-related diseases (López-Otín et al., 2023). In aged mice and renal epithelial cell models, PQQ supplementation significantly reduced the levels of inflammatory markers, including interleukin 6 (IL-6) and tumor necrosis factor alpha (TNF-α), primarily through inhibition of NF-κB-related signaling (Wang et al., 2019; Mohamad Ishak et al., 2024). These findings suggest that PQQ attenuates age-associated inflammatory signaling and helps restore immune balance and intercellular communication during aging.

SPD exhibits multifaceted anti-inflammatory effects across chronic inflammatory contexts. In macrophages, SPD suppressed pro-inflammatory (M1-like) polarization and promoted anti-inflammatory (M2-like) polarization (Liu et al., 2020), thereby alleviating adipose tissue inflammation, hepatic steatosis, and osteoarthritic inflammation in experimental models (Ma et al., 2021; Ou et al., 2024). In enteritis/colitis models, SPD reduced tissue inflammation in association with improved intestinal homeostasis (Niechcial et al., 2023). Within the central nervous system, SPD reduced neuroinflammation and amyloid-β burden in neurodegeneration-relevant models (Xu et al., 2020; Freitag et al., 2022). These findings indicate that SPD mitigates chronic inflammation across multiple organs through shared mechanisms involving autophagy induction and immune homeostasis restoration.

### 3.12 Dysbiosis: modulation of gut–immune–inflammatory axis

Age-related gut dysbiosis contributes to systemic inflammation and metabolic dysfunction by disrupting the gut–immune axis (López-Otín et al., 2023). In piglet models, dietary PQQ reversed pathogen-induced dysbiosis by increasing beneficial *Lactobacillus* populations, normalizing the production of short-chain fatty acids (SCFAs), particularly butyrate, and improving mucosal immune function (Huang et al., 2020). These effects were accompanied by reduced inflammatory signaling and enhanced antioxidant defenses, suggesting that PQQ may indirectly influence aging-related processes through modulation of the gut–immune axis (Huang et al., 2020).

SPD suppresses the progression of chronic inflammatory and metabolic disorders in mouse models by strengthening intestinal barrier function and promoting normalization of gut microbiota (Ma et al., 2020; Liu et al., 2022; Niechcial et al., 2023). In diet-induced obesity and colitis models, SPD reduced inflammatory cytokines and circulating endotoxin-related molecules (e.g., LPS) while improving barrier integrity and enriching beneficial bacterial taxa, including SCFA-producing groups (Ma et al., 2020; Niechcial et al., 2023). In an experimental abdominal aortic aneurysm model, SPD altered gut microbiota composition, consistent with a gut–vascular inflammatory link (Liu et al., 2022). Collectively, these findings suggest coordinated regulation of chronic inflammation through interactions between gut microbiota, immune responses, and host metabolism.

## 4 Discussion

Our review indicated that the roles of PQQ and SPD intersect with multiple hallmarks of aging through partially overlapping regulatory networks. Diverse upstream signals often converge on conserved integrator nodes (e.g., AMPK, sirtuins, mTOR/autophagy) that coordinate energy balance, mitochondrial function, and cellular maintenance. In this framework, PQQ is primarily a redox-active mitochondrial modulator, whereas SPD is more closely linked to polyamine biology and autophagy-dependent cellular housekeeping and nutrient-state (Jonscher et al., 2021; Hofer et al., 2024; Yan et al., 2024). Therefore, their “complementarity” is represented by different entry points into partially shared networks; however, whether combined supplementation is additive or context-dependent requires empirical analysis.

Aging reflects progressive dysregulation across interconnected systems rather than single defects (Feng et al., 2026). The relevance of PQQ and SPD lies in their potential to influence converging processes related to mitochondrial dysfunction, loss of proteostasis, inflammaging, and cellular senescence. However, the strength and translational maturity of evidence vary across models and endpoints, and neutral effects are plausible depending on baseline status, tissue context, dose, and outcome selection. Recent system-level evidence suggests that immune-aging phenotypes may be measurable targets for nutritional interventions (Dugan et al., 2023). Together, these observations support targeting complementary entry points within interconnected aging networks for achieving more robust outcomes than single-pathway interventions.

### 4.1 Translational barriers

Despite compelling mechanistic evidence, the clinical translation of PQQ and SPD remains preliminary. Most data are derived from *in vitro* systems or short-lived model organisms and may not represent human aging. Interpretation is further constrained by reliance on surrogate biomarkers and the limited use of standardized aging measures (López-Otín et al., 2023). A major barrier is dose realism: many preclinical exposures exceed what is achievable through diet or typical supplements, complicating mechanism and efficacy extrapolation (Yan et al., 2024). Dose–response studies, pharmacokinetic/pharmacodynamic bridging, and target-engagement biomarkers are needed to define clinically realistic regimens.

### 4.2 Safety considerations

For SPD, emerging evidence suggests context-dependent cardiovascular risks. Elevated circulating SPD has been associated with increased stroke risk in some populations (Heshmati and Jarisch, 2025), and genotype-dependent effects on lifespan have been reported (Su et al., 2025). These findings highlight the need for cardiovascular risk assessment and potential genetic stratification prior to supplementation.

For PQQ, uncertainties in human pharmacokinetics limit understanding of tissue exposure. As a fermentation-derived compound, it may carry theoretical allergenicity risks. Regulatory assessments exclude pregnant and lactating populations without clear mechanistic rationale (Turck et al., 2017). While short-term studies indicate good tolerability, long-term safety at higher doses remains unclear (Nakano et al., 2014, Shiojima et al., 2022b).

### 4.3 Future directions and recommendations

Stronger evidence is needed regarding long-term safety and potential interactions. SPD appears generally well tolerated, however, long-term data and evidence in high-risk populations are limited (Schwarz et al., 2018; 2022; Keohane et al., 2024). PQQ is considered safe at typical intake levels, with short-term tolerability at ∼20 mg/day (Turck et al., 2017; Nakano et al., 2014; Shiojima et al., 2022a), but long-term data are lacking. Notably, safety data for combined PQQ and SPD supplementation are absent. Moreover, potential interactions with medications, particularly those affecting redox balance, mitochondrial function, or polyamine metabolism, remain insufficiently characterized.

Future clinical trials should include explicit monitoring for cardiovascular endpoints (arrhythmia, blood pressure, thromboembolism), allergic reactions, and population-specific toxicity signals, with baseline stratification by cardiovascular risk, genetic background, and comorbidity burden. Furthermore, future research should prioritize long-term randomized trials with functional endpoints, standardized aging biomarkers (e.g., SASP, epigenetic clocks), and rigorous safety monitoring. Combined PQQ and SPD interventions should be evaluated to determine whether complementary pathway targeting improves health span.

## 5 Conclusion

PQQ and SPD are biologically grounded nutritional candidates that target conserved aging pathways, particularly mitochondrial biogenesis/function and autophagy-linked quality control. While current evidence highlights plausible mechanisms, translation is limited by dosing gaps, endpoint heterogeneity, and limited long-term safety data. Biomarker-guided long-duration human studies are needed to define effective dosing, safety margins, and optimal use, including combination strategies for healthy longevity.
