# Supplementary material for: Dietary pyrroloquinoline quinone and spermidine in healthy longevity: targeting the hallmarks of aging
Source: Front Aging. 2026 May 15;7:1791853. doi: 10.3389/fragi.2026.1791853 (PMC13219961; doi:10.3389/fragi.2026.1791853)
Supplement: Supplementary file 1 [file Table1.docx]

Supplementary Material

# Literature Search Strategy

A structured literature search was conducted to identify relevant studies examining pyrroloquinoline quinone (PQQ) and spermidine (SPD) in the context of aging biology. Searches were performed in PubMed, Scopus, and Web of Science databases covering publications from January 2020 to December 2025. Search terms included combinations of: “Pyrroloquinoline quinone” OR “PQQ” OR AND “aging” OR “mitochondria” OR “oxidative stress” OR “cellular senescence,” and “spermidine” AND “aging” OR “autophagy” OR “longevity.” Other related terms from hallmarks of aging such as “DNA damage”, “telomeres shortening”, “lifespan” was also included in the search.

Only peer-reviewed original research articles and review articles published in English were considered. Conference abstracts, editorials, and non-English publications were excluded. Studies were selected based on relevance to the hallmarks of aging framework, with particular emphasis on mechanistic insights into mitochondrial function, proteostasis, autophagy, oxidative stress, and related pathways.

# Supplementary Data

Table S1: Summary of evidence linking PQQ and SPD to key hallmarks of aging and underlying mechanisms.

| Hallmarks | Experimental model | Key findings | Mechanisms | References |
| --- | --- | --- | --- | --- |
| Genomic instability | In vivo  Bmi-1 knockout mice | PQQ reduces oxidative DNA damage | PQQ lowers ROS levels and reduces 8-OHdG, a marker of DNA damage. It also helps the cells repair DNA more effectively, improving overall genomic protection. | (Li *et al*., 2022) |
|  | In vivo  (OSCC model mice) | SPD suppresses oral carcinogenesis through autophagy induction. | Spermidine promotes the expression of proteins involved in DNA repair and cellular stress response. | (Coeli-Lacchini *et al*., 2023) |
| Telomere attrition | In vivo  (C57Bl/6JRj mice) | Spermidine promotes cardio-protective effects. | Spermidine treatment protects against telomeres shortening in cardiac tissue | (Wirth *et al*., 2021) |
| Epigenetic alterations | In vivo  human B-cell lymphoma models | PQQ has been found to interact with epigenetic regulators which is closely linked to cancer and developmental disorders. | PQQ can inhibit PRC2 (EZH2) methyltransferase activity, and PQQ alters sirtuin (SIRT1/SIRT3) expression/activity which has downstream epigenetic consequences. | (M. Jiang *et al*., 2025) |
|  | In vivo  (liver-specific IRS1 KO mice) | Spermidine contributes to lifespan extension by reprogramming gene expression profiles through epigenetic regulation. | Reduced insulin signaling boosts GNMT activity, increasing spermidine production.Spermidine is essential for lifespan extension under low insulin signaling. | (Tain *et al*., 2020) |
|  | In vivo  (Honey bees) | Spermidine contributes to increased honeybee longevity. | Spermidine induces epigenetic changes and promotes the expression of autophagy-related genes. | (Kojić *et al*., 2024) |
|  | In vivo  (NAT1-deficient mice)  Clinical | Spermidine supplementation decreases RIPK1-mediated cell death and diabetic phenotypes induced by NAT1 deficiency in vivo. | Spermidine suppresses inflammation through acetylhypusination of RIPK1. | (Zhang *et al*., 2024) |
| Loss of proteostasis | In vivo  (C57BL/6N male mice) | Yeast-fermented garlic extract containing polyamines activated autophagy | mTORC1-independent, EGR1-dependent pathway. | (Xie K *et al*., 2025) |
| Disabled macroautophagy | In-vivo  Caenorhabditis elegans | PQQ extends lifespan and activates autophagy | PQQ increased expression of autophagy-related genes (including lgg-1 and bec-1) and enhanced autophagic structures (GFP::LGG-1 puncta). This autophagy activation is linked to insulin/IGF1 signaling and is required for PQQ’s pro-longevity effects. | (Yang *et al*., 2021) |
|  | In vitro  HAPI microglia cells treated with lipopolysaccharide (LPS) | PQQ increases autophagy in microglial cells | PQQ increases autophagy markers (LC3, LAMP2) and lysosomal activity in microglial cells exposed to inflammatory stress. PQQ also Changes in autophagy dynamics through the PI3K/Akt signaling pathway and Enhanced autophagy correlates with reduced apoptosis and stress responses in microglia. | (Gao *et al*., 2021) |
|  | BV2 microglia cells with rotenone injury | PQQ enhances autophagy in models of neuroinflammation | PQQ increased autophagosome markers (LC3-II, Atg5), enhanced mitochondria trafficking to lysosomes. PQQ activated mitophagy pathways (e.g., PINK1/Parkin). Autophagy is required for the anti-inflammatory effect of PQQ | (Zhang *et al*., 2020) |
|  | In vivo  (Drosophila, Mouse, human) | Spermidine improves cognitive function. | The beneficial effects of SPD appear to depend on autophagy- and likely mitophagy-related processes. | (Schroeder *et al.*, 2021) |
|  | In vivo  (Human) | Spermidine intake is associated with cortical thickness and hippocampal volume in older adults | Spermidine may contribute to brain health by inhibiting acetyltransferases and inducing autophagy | (Schwarz *et al*., 2020) |
|  | In vivo  (Human) | Spermidine improved cognitive performance in subjects with mild and moderate dementia. | Spermidine has the ability to trigger the important process of dissolving amyloid-beta plaques by autophagy. | (Pekar *et al.*, 2021) |
| Deregulated nutrient-sensing | In vivo  L6 myotube muscle cells | PQQ increases AMPK and other metabolic regulators in muscle cells | PQQ increased protein levels of phosphorylated AMPK, PGC-1α, and SIRT1 in metabolic stress induced cell, indicating activation of both energy sensing and mitochondrial regulation pathways. These pathways are core components of the nutrient-sensing network and tie into metabolic homeostasis. | (Supruniuk *et al.*, 2020) |
|  | In vivo  Natural aged mice | In aging mice, PQQ supplementation prevents abnormal fat loss by improving nutrient-sensing pathways and metabolic efficiency, indicating better preservation of adipose tissue function during aging. | Not fully established; likely related to AMPK–Sirtuin–mitochondrial pathways. | (Mohamad Ishak *et al*., 2024) |
|  | In vivo  Caenorhabditis elegans | PQQ’s lifespan-extending effects involved the insulin/IGF1 signaling pathway (IIS) | PQQ improves insulin/IGF-1 signaling | (Yang *et al*., 2021) |
|  | In vivo  (wild-type mice) | Spermidine protects against metabolic dysfunction during overnutrition. | The metabolic benefits of spermidine are independent of autophagy in adipose tissue. | (Liao *et al*., 2021) |
|  | In vivo  (Drosophila, Warm) | Spermidine is essential for fasting-mediated autophagy and longevity | Spermidine mediated these effects via autophagy induction and hypusination of the translation regulator eIF5A. | (Hofer *et al*., 2024) |
| Mitochondrial dysfunction | In vivo  POI model mice | Combined treatment of mesenchymal stem cell-derived mitochondria (MSC-Mito) with PQQ promotes mitochondrial biogenesis. | PQQ increases the protein expression of SIRT1 and PGC-1α. | (Liu *et al*., 2024) |
|  | In vitro  HEI-OC1 cells | PQQ inhibits premature aging of auditory cells caused by oxidative stress. | PQQ promotes the regulation of SIRT1/PGC-1α, SIRT1 protein expression, PGC-1α deacetylation, and the recovery of ATP production rate and mitochondrial biogenesis. | (Gao *et al*., 2022) |
|  | In vitro  HAPI microglia | PQQ reduces apoptosis by increasing autophagy activity. | PQQ alters the intracellular distribution of LAMP2 via the PI3K/Akt signaling pathway. | (Gao *et al.*, 2021) |
|  | In vivo  (aged rats) | SPD increases antioxidation mechanisms, maintains mitochondrial homeostasis, and delays cardiac aging. | Activating SIRT1 promotes the deacetylation of PGC-1α and enhances mitochondrial biogenesis. | (Wang *et al*., 2020) |
|  | In vivo  (aged mice) | Spermidine supplementation improves mitochondrial number and morphology in the hearts of aged mice. | Spermidine counteracts age-related reductions and structural abnormalities of mitochondria, thereby preserving mitochondrial homeostasis. | (Messerer *et al*., 2023) |
|  | In vivo  (aged mice) | Spermidine improved the declined angiogenic capacity of senescent ECs, including migration and tube-formation. | Spermidine promotes autophagy and mitophagy, and improved mitochondrial quality in senescent ECs | (Ueno *et al*., 2023) |
| Cellular senescence | In vivo  Bmi1 heterozygous (Bmi1) mice | PQQ inhibits skin aging. | PQQ prevents DNA damage by reducing oxidative stress and inhibits matrix metalloproteinases (MMPs). | (Li *et al*., 2022) |
|  | In vitro  KGN cells | Combined treatment of mesenchymal stem cell-derived mitochondria (MSC-Mito) and PQQ inhibits DNA damage and reduces cell apoptosis caused by DNA damage. | PQQ upregulates SIRT1 gene expression, thereby suppressing activation of the ATM/p53 signaling pathway. | (Liu *et al*., 2024) |
|  | In vitro  Human prostate stromal cell line (PSC27) | PQQ down-regulates the expression of the senescence-associated secretory phenotype (SASP). | PQQ targets the chaperone HSPA8 and disrupts the activation of downstream signaling pathways, including the p38/Akt/mTOR and NF-κB complex-mediated axes. | (B. Jiang *et al*., 2025) |
|  | In vivo  mice | PQQ has anti-apoptotic and anti-aging properties and plays a role in promoting immune regeneration. | PQQ suppresses the expression of the apoptosis-related marker ASPP1 and suppresses the age-related decline in CD34 and CD62L levels. | (Liu *et al*., 2025) |
|  | In vivo  Mice | PQQ prevents bone loss and aging due to age. | PQQ inhibits oxidative stress and osteoclast activation via Nrf2. | (Li *et al*., 2023) |
|  | In vitro  hBM‐MSCs | PQQ inhibits bone cell aging. | PQQ binds to MCM3, stabilizing it, and MCM3 competes with Nrf2 to bind to Keap1, enhancing Nrf2-ARE signaling. | (Li *et al*., 2023) |
|  | In vitro: D-gal-induced C2C12 myoblast cells | PQQ decreases age-related accumulation of senescent cells and helps preserve cellular function in aged models | PQQ mitigates the pro-senescence signaling cascade that arises from oxidative stress | (Mohamad Ishak *et al*., 2024) |
|  | In vivo  (Ovarian oxidative stress model mice) | Spermidine alleviates ovarian damage by suppressing oxidative stress and ferroptosis. | Spermidine activates the Nrf2/HO-1/GPX4 axis to reduce lipid peroxidation and ROS. It also modulates the Akt/FHC/ACSL4 pathway to inhibit ferroptosis. | (Niu *et al*., 2023) |
|  | In vivo  (mice) | Spermidine alleviates thymopoiesis defects and aging of the peripheral T-cell population in mice after radiation exposure. | SPD induces autophagy in T cells and helps maintain immune system balance. | (Yoshida *et al*., 2025) |
|  | In vivo  (natural aging rat) | Spermidine supplementation improves cellular redox balance and ionic homeostasis in D-galactose induced and naturally aged rat models. | Spermidine reduces age-related increases in reactive oxygen species, lipid and protein oxidation while boosting antioxidant levels, thereby protecting cellular redox status. | (Singh *et al*., 2025) |
| Stem cell exhaustion | In vivo  (SC-specific *eIF5A*-KO and *Myod*-KO mice) | Spermidine is involved in satellite cell activation, muscle regeneration, and the regulation of aging | Spermidine-eIF5A axis activates QSCs by selectively translating the Myod mRNA. | (Zhang *et al*., 2024) |
|  | In vivo  (DP animal model) | Spermidine alleviates diabetic periodontitis by reversing senescence of human periodontal ligament stem cells via mitophagy. | Spermidine activates mitophagy to restore mitochondrial function and reduce oxidative stress, thereby suppressing cGAS-STING–dependent senescence of periodontal ligament stem cells. | (Zhou *et al*., 2025) |
|  | In vivo  (Male Lewis rats) | Novel benefits of polyamines in intestinal adaptation for SBS and associated impaired liver disorder. | Spermidine improves intestinal immune function, barrier function and liver function. | (Kasahara *et al*., 2024) |
| Altered intercellular communication | In vivo  (Human) | SPD contributes to neural development and the improvement of cellular homeostasis. | Spermidine has been shown to restore autophagy impaired by defects in cellular transport. | (Díaz-Osorio *et al*., 2025) |
|  | In vivo  (Middle-aged male rats) | Early treatment with spermidine effectively delayed the senescence of mesenchymal stem cells (MSCs). | SPD prevents MSC senescence by reducing oxidative stress through SIRT3. | (Huang *et al*., 2023) |
| Chronic inflammation | In vivo: natural aged mice  In vivo: HK-2 cells | PQQ exhibits anti-inflammatory action. | PQQ reduces inflammatory responses by lowering the levels of IL-6, TNF-α, and CRP, and by inhibiting activation of the NF-κB signaling pathway. | (Mohamad Ishak *et al*., 2024; Wang *et al*., 2019) |
|  | In vivo  （SAMP8 mice） | Spermidine improved cognitive function in SAMP8 mice. | Spermidine suppressed age-related neuronal changes by inducing autophagy and restoring mitochondrial homeostasis. | (Xu *et al*., 2020) |
|  | In vivo  （OA Mouse） | Spermidine ameliorated cartilage degeneration and synovitis. | Spermidine promoted M2 polarization of macrophages, thereby preventing cartilage degradation and chondrocyte death. | (Ou *et al.*, 2024) |
|  | In vivo  （HDF Mouse） | Spermidine reduced hepatic steatosis and improved plasma lipid profiles. | Spermidine suppressed inflammation in adipose tissue and improved intestinal barrier function. | (Ma *et al*., 2021) |
| Dysbiosis | In vivo  piglets | PQQ regulates gut microbiota and reduces inflammatory injury | PQQ reversed dysbiosis by attenuating pathogen-induced changes in bacterial communities, increasing beneficial Lactobacillus, and normalizing short-chain fatty acids like butyrate. PQQ also improved mucosal immunity and antioxidant enzymes and reduced NF-κB-mediated inflammation and mucosal damage. | (Huang *et al*., 2020) |
|  | In vivo (DIO Mouse) | Spermidine increased short-chain fatty acid (SCFA)–producing bacteria, thereby improving metabolism and intestinal barrier function. | Spermidine improved intestinal barrier integrity and reduced inflammation through epithelial autophagy and modulation of the gut microbiota. | (Ma *et al*., 2020) |
|  | In vivo  （T cell transfer colitis model mice） | Spermidine promotes colitis-protective and anti-inflammatory effects via PTPN2. | Spermidine induced macrophage M2 polarization via PTPN2-mediated signaling, prevented pathological overgrowth of Proteobacteria, and maintained a healthy gut microbiota. | (Niechcial *et al*., 2023) |
|  | In vivo  （AAA Mice） | Spermidine suppressed the expansion rate of abdominal aortic aneurysms (AAA). | By improving the gut microbiota and its metabolic functions, spermidine reduced systemic and vascular local chronic inflammation and structural degradation, thereby inhibiting AAA progression. | (Liu *et al*., 2022) |

# References

Coeli-Lacchini, F.B., da Silva, G., Belentani, M., Alves, J.S.F., Ushida, T.R., Lunardelli, G.T., *et al*. (2023). spermidine suppresses oral carcinogenesis through autophagy induction, DNA damage repair, and oxidative stress reduction. *American Journal of Pathology* *193*(12), 2172–2181. doi: 10.1016/j.ajpath.2023.09.005

Díaz‐Osorio, Y., Gimeno‐Agud, H., Mari‐Vico, R., Illescas, S., Ramos, J.M., Darling, A., *et al*. (2025). Spermidine recovers the autophagy defects underlying the pathophysiology of cell trafficking disorders. *Journal of Inherited Metabolic Disease* *48*(1), e12841. doi: 10.1002/jimd.12841

Gao, S., Zhou, Q., Jin, H., Shi, N., Wang, X., Zhang, L., and Yan, M. (2021). Effect of pyrroloquinoline quinone on lipopolysaccharide-induced autophagy in HAPI microglial cells. *Annals of Translational Medicine* *9*(17), 1377–1377. doi: 10.21037/atm-21-730

Gao, Y., Kamogashira, T., Fujimoto, C., Iwasaki, S., and Yamasoba, T. (2022). Pyrroloquinoline quinone (PQQ) protects mitochondrial function of HEI-OC1 cells under premature senescence. *Npj Aging 8*(1), 3. doi: 10.1038/s41514-022-00083-0

Hofer, S.J., Daskalaki, I., Bergmann, M., Friščić, J., Zimmermann, A., Mueller, M.I., *et al*. (2024). Spermidine is essential for fasting-mediated autophagy and longevity. *Nature Cell Biology 26*(9), 1571–1584. doi: 10.1038/s41556-024-01468-x

Huang, C., Ming, D., Wang, W., Wang, Z., Hu, Y., Ma, X., and Wang, F. (2020). Pyrroloquinoline quinone alleviates jejunal mucosal barrier function damage and regulates colonic microbiota in piglets challenged with enterotoxigenic Escherichia coli. *Frontiers in Microbiology 11*, 1754. doi: 10.3389/fmicb.2020.01754

Huang, H., Zhang, W., Su, J., Zhou, B., and Han, Q. (2023). Spermidine retarded the senescence of multipotent mesenchymal stromal cells in vitro and in vivo through SIRT3-mediated antioxidation. *Stem Cells International 2023*, 1–17. doi: 10.1155/2023/9672658

Jiang, B., Zhang, H., Xu, Q., Jiang, Z., He, R., Fu, Q., and Sun, Y. (2025). Pyrroloquinoline quinone is an effective senomorphic agent to target the pro‐inflammatory phenotype of senescent cells. *Aging Cell 24*(9), e70138. doi: 10.1111/acel.70138

Jiang, M., Huang, F., Hong, X., Xu, C., Zhang, B., Hu, S., *et al*. (2025). PQQ Inhibits PRC2 methyltransferase activity and suppresses the proliferation of B-cell lymphoma in vitro. *Chemistry & Biodiversity 22*(7), e202500198. doi: 10.1002/cbdv.202500198

Kasahara, N., Teratani, T., Yokota, S., Sakuma, Y., Sasanuma, H., Fujimoto, Y., *et al*. (2024). Dietary polyamines promote intestinal adaptation in an experimental model of short bowel syndrome. *Scientific Reports 14*(1), 4605. doi: 10.1038/s41598-024-55258-4

Kojić, D., Spremo, J., Đorđievski, S., Čelić, T., Vukašinović, E., Pihler, I., and Purać, J. (2024). Spermidine supplementation in honey bees: Autophagy and epigenetic modifications. *PLOS ONE 19*(7), e0306430. doi: 10.1371/journal.pone.0306430

Li, J., Liu, M., Liang, S., Yu, Y., and Gu, M. (2022). Repression of the antioxidant pyrroloquinoline quinone in skin aging induced by Bmi-1 deficiency. *BioMed Research International* *2022*, 1–12. doi: 10.1155/2022/1732438

Li, J., Zhang, J., Xue, Q., Liu, B., Qin, R., Li, Y., *et al.* (2023). Pyrroloquinoline quinone alleviates natural aging‐related osteoporosis via a novel MCM3‐Keap1‐Nrf2 axis‐mediated stress response and Fbn1 upregulation. *Aging Cell 22*(9), e13912. doi: 10.1111/acel.13912

Liao, C.-Y., Kummert, O.M.P., Bair, A.M., Alavi, N., Alavi, J., Miller, D.M., *et al*. (2021). The autophagy inducer spermidine protects against metabolic dysfunction during overnutrition. *Journals of Gerontology: Series A 76*(10), 1714–1725. doi: 10.1093/gerona/glab145

Liu, S., Liu, Y., Zhao, J., Yang, P., Wang, W., & Liao, M. (2022). Effects of spermidine on gut microbiota modulation in experimental abdominal aortic aneurysm mice. *Nutrients 14*(16), 3349. doi: 10.3390/nu14163349

Liu, S., Wang, Y., Yang, H., Tan, J., Zhang, J., and Zi, D. (2024). Pyrroloquinoline quinone promotes human mesenchymal stem cell-derived mitochondria to improve premature ovarian insufficiency in mice through the SIRT1/ATM/p53 pathway. *Stem Cell Research & Therapy 15*(1), 97. doi: 10.1186/s13287-024-03705-4

Liu, X., Zhang, C., Lv, J., Liu, Y., Gu, C., Gao, Y., *et al*. (2025). Pyrroloquinoline Quinone Reprograms the Single-Cell Landscape of Immune Aging in Hematopoietic Immune System. *Aging Cell*, 2025; 24:e70050. doi: 10.1111/acel.70050

Ma, L., Ni, Y., Hu, L., Zhao, Y., Zheng, L., Yang, S., *et al*. (2021). Spermidine ameliorates high-fat diet-induced hepatic steatosis and adipose tissue inflammation in preexisting obese mice. *Life Sciences 265*, 118739. doi: 10.1016/j.lfs.2020.118739

Ma, L., Ni, Y., Wang, Z., Tu, W., Ni, L., Zhuge, F., *et al.* (2020). Spermidine improves gut barrier integrity and gut microbiota function in diet-induced obese mice. *Gut Microbes 12*(1), 1832857. doi: 10.1080/19490976.2020.1832857

Messerer, J., Wrede, C., Schipke, J., Brandenberger, C., Abdellatif, M., Eisenberg, T. (2023). Spermidine supplementation influences mitochondrial number and morphology in the heart of aged mice. *Journal of Anatomy 242*(1), 91–101. doi: 10.1111/joa.13618

Mohamad Ishak, N.S., Kikuchi, M., and Ikemoto, K. (2024). Dietary pyrroloquinoline quinone hinders aging progression in male mice and D-galactose-induced cells. *Frontiers in Aging 5*, 1351860. doi: 10.3389/fragi.2024.1351860

Niechcial, A., Schwarzfischer, M., Wawrzyniak, M., Atrott, K., Laimbacher, A., Morsy, Y., *et al*. (2023). Spermidine ameliorates colitis via induction of anti-inflammatory macrophages and prevention of intestinal dysbiosis. *Journal of Crohn’s and Colitis 17*(9), 1489–1503. doi: 10.1093/ecco-jcc/jjad058

Niu, C., Jiang, D., Guo, Y., Wang, Z., Sun, Q., Wang, X., *et al*. (2023). Spermidine suppresses oxidative stress and ferroptosis by Nrf2/HO-1/GPX4 and Akt/FHC/ACSL4 pathway to alleviate ovarian damage. *Life Sciences 332*, 122109. doi: 10.1016/j.lfs.2023.122109

Ou, Q., Tang, S., Zhu, J., Xue, S., Huang, H., Zhao, Y., *et al.* (2024). Spermidine ameliorates osteoarthritis via altering macrophage polarization. *Biochimica et Biophysica Acta (BBA) Molecular Basis of Disease*, *1870*(4), 167083. doi: 10.1016/j.bbadis.2024.167083

Pekar, T., Bruckner, K., Pauschenwein-Frantsich, S., Gschaider, A., Oppliger, M., Willesberger, J., *et al*. (2021). The positive effect of spermidine in older adults suffering from dementia. *Wiener Klinische Wochenschrift 133*(9–10), 484–491. doi: 10.1007/s00508-020-01758-y

Schroeder, S., Hofer, S.J., Zimmermann, A., Pechlaner, R., Dammbrueck, C., Pendl, T., *et al*. (2021). Dietary spermidine improves cognitive function. *Cell Reports 35*(2), 108985. doi: 10.1016/j.celrep.2021.108985

Schwarz, C., Horn, N., Benson, G., Wrachtrup-Calzado, I., Wurdack, K., Pechlaner, R., *et al*. (2020). Spermidine intake is associated with cortical thickness and hippocampal volume in older adults. *NeuroImage 221*, 117132. doi: 10.1016/j.neuroimage.2020.117132

Singh, S., Verma, A.K., Garg, G., Singh, A.K., and Rizvi, S.I. (2025). Spermidine protects cellular redox status and ionic homeostasis in D-galactose-induced senescence and natural aging rat models. *Zeitschrift für Naturforschung C 80*(5–6), 285–295. doi: 10.1515/znc-2024-0181

Supruniuk, E., Mikłosz, A., and Chabowski, A. (2020). Pyrroloquinoline quinone modifies lipid profile, but not insulin sensitivity, of palmitic acid-treated L6 myotubes. *International Journal of Molecular Sciences 21*(21), 8382. doi: 10.3390/ijms21218382

Tain, L.S., Jain, C., Nespital, T., Froehlich, J., Hinze, Y., Grönke, S., *et al*. (2020). Longevity in response to lowered insulin signaling requires glycine N-methyltransferase-dependent spermidine production. *Aging Cell 19*(1), e13043. doi: 10.1111/acel.13043

Ueno, D., Ikeda, K., Yamazaki, E., Katayama, A., Urata, R., and Matoba, S. (2023). Spermidine improves angiogenic capacity of senescent endothelial cells, and enhances ischemia-induced neovascularization in aged mice. *Scientific Reports 13*(1), 8338. doi: 10.1038/s41598-023-35447-3

Wang, J., Li, S., Wang, J., Wu, F., Chen, Y., Zhang, H., *et al*. (2020). Spermidine alleviates cardiac aging by improving mitochondrial biogenesis and function. *Aging 12*(1), 650–671. doi: 10.18632/aging.102647

Wang, Z., Han, N., Zhao, K., Li, Y., Chi, Y., and Wang, B. (2019). Protective effects of pyrroloquinoline quinone against oxidative stress-induced cellular senescence and inflammation in human renal tubular epithelial cells via Keap1/Nrf2 signaling pathway. *International Immunopharmacology 72*, 445–453. doi: 10.1016/j.intimp.2019.04.040

Wirth, A., Wolf, B., Huang, C.-K., Glage, S., Hofer, S.J., Bankstahl, M., *et al*. (2021). Novel aspects of age-protection by spermidine supplementation are associated with preserved telomere length. *GeroScience 43*(2), 673–690. doi: 10.1007/s11357-020-00310-0

Xu, T.-T., Li, H., Dai, Z., Lau, G.K., Li, B.-Y., Zhu, W.-L., *et al*. (2020). Spermidine and spermine delay brain aging by inducing autophagy in SAMP8 mice. *Aging 12*(7), 6401–6414. doi: 10.18632/aging.103035

Yang, J., Zhang, C., Li, Z., Gao, Y., Jiang, L., Zhang, J., *et al*. (2025). Spermine alleviates myocardial cell aging by inhibiting mitochondrial oxidative stress damage. *European Journal of Pharmacology 997*, 177477. doi: 10.1016/j.ejphar.2025.177477

Yang, L., Ye, Q., Zhang, X., Li, K., Liang, X., Wang, M., *et al*. (2021). Pyrroloquinoline quinone extends Caenorhabditis elegans’ longevity through the insulin/IGF1 signaling pathway-mediated activation of autophagy. *Food & Function 12*(22), 11319–11330. doi: 10.1039/D1FO02128A

Yang, X., Zhang, M., Dai, Y., Sun, Y., Aman, Y., Xu, Y., *et al*. (2020). Spermidine inhibits neurodegeneration and delays aging via the PINK1-PDR1-dependent mitophagy pathway in C. elegans. *Aging 12*(17), 16852–16866. doi: 10.18632/aging.103578

Yoshida, K., Liu, Z., Kubo, Y., Miura, M., Yamaoka, M., Nagamura, H., *et al.* (2025). Spermidine alleviates thymopoiesis defects and aging of the peripheral T-cell population in mice after radiation exposure. *Experimental Gerontology*, *199*, 112646. doi: 10.1016/j.exger.2024.112646

Zhang, Q., Han, W., Wu, R., Deng, S., Meng, J., Yang, Y., *et al*. (2024). Spermidine-eIF5A axis is essential for muscle stem cell activation via translational control. *Cell Discovery 10*(1), 94. doi: 10.1038/s41421-024-00712-w

Zhang, T., Fu, W., Zhang, H., Li, J., Xing, B., Cai, Y., *et al*. (2024). Spermidine mediates acetylhypusination of RIPK1 to suppress diabetes onset and progression. *Nature Cell Biology 26*(12), 2099–2114. doi: 10.1038/s41556-024-01540-6

Zhou, Y., Wang, D., Xiao, Q., Ma, L., Gou, H., Ru, Y., *et al*. (2025). Spermidine alleviates diabetic periodontitis by reversing human periodontal ligament stem cell senescence via mitophagy. Free *Radical Biology and Medicine*, 227, 379–394. doi: 10.1016/j.freeradbiomed.2024.12.016
